# Quantitative analysis of hormones and inflammatory cytokines in *Chlamydia trachomatis*-infected women with tubal ectopic pregnancy and early intrauterine pregnancy

**DOI:** 10.1016/j.dib.2015.11.048

**Published:** 2015-12-02

**Authors:** Ruijin Shao, Yi Feng, Shien Zou, Xin Li, Peng Cui, Håkan Billig

**Affiliations:** a Department of Physiology/Endocrinology, Institute of Neuroscience and Physiology, The Sahlgrenska Academy at University of Gothenburg, Gothenburg 40530, Sweden; b Department of Integrative Medicine and Neurobiology, State Key Laboratory of Medical Neurobiology, Shanghai Medical College; Institute of Acupuncture Research (WHO Collaborating Center for Traditional Medicine); Institutes of Brain Science, Fudan University, Shanghai 200032, China; c Department of Gynecology, Obstetrics and Gynecology Hospital of Fudan University, Shanghai 200011, China; d Shanghai Key Laboratory of Female Reproductive Endocrine Related Diseases, 200011 Shanghai, China

**Keywords:** Chlamydia trachomatis infection, Cytokines, Intrauterine pregnancy, Ectopic pregnancy

## Abstract

In this data, non-pregnant women during the menstrual cycle, women with normal intrauterine pregnancy (IUP), and women with tubal ectopic pregnancy (EP) after informed consent were included. The serum levels of 17β-estradiol, progesterone, testosterone, beta-human chorionic gonadotropin, interleukin (IL)-1β, IL-4, IL-6, IL-7, IL-8, IL-10, tumor necrosis factor α (TNFα), and interferon-γ (IFN-γ), epidermal growth factor, the *Chlamydia (C.) trachomatis* IgG and HSP60 were analyzed. Receiver operating characteristic analysis was used to assess the diagnostic discrimination of tubal EP and gestational age-matched IUP. Our data show that *C. trachomatis* infection is associated with IL-8 levels, which had excellent discriminative validity in positively identifying tubal EP (concomitant with *C. trachomatis* infection) from IUP and non-pregnant conditions regardless of *C. trachomatis* infection.

**Specifications Table**

**Table:** 

Subject area	Biomedicine
More specific subject area	Ectopic pregnancy in humans, clinical biomarkers
Type of data	Tables and figure
How data was acquired	Competitive RIA, human cytokine magnetic bead array and ELISA kits
Data format	Analyzed and graphed
Experimental factors	A total of 225 blood samples were obtained (139 in the non-pregnant menstrual cycle group, 50 in the early intrauterine pregnancy (IUP) group, and 36 in the tubal EP group). Clinical information on the patients was collected and entered into a dedicated database. The serum levels of 17β-estradiol, progesterone, testosterone, beta-human chorionic gonadotropin were measured by competitive RIA using direct-coated tube technology. The serum levels of IL-1β, IL-4, IL-6, IL-7, IL-8, IL-10, tumor necrosis factor α (TNFα), and interferon-γ (IFN-γ) cytokines were quantified by high sensitivity human cytokine magnetic bead array.
Experimental features	1. Epidermal growth factor (EGF), a marker of trophoblast invasion and placental development, was measured. 2. The *C. trachomatis* IgG and HSP60 human ELISA kits were used for screening and diagnosis of *C. trachomatis* infection. 3. Receiver operating characteristic (ROC) curves were used to assess the specificity and sensitivity of the cytokine levels in response to *C. trachomatis* infection for discriminating tubal EP from IUP and non-pregnant conditions.
Data source location	Shanghai, China
Data accessibility	Data are available with this article

## Value of the data

1. The data indicate that using a combination of serum IL-8 and steroid hormone levels can possibly differentiate a certain group of women with tubal EP from those with normal early IUP.
2. The data are useful for dissecting the molecular mechanism of different cytokines in the Fallopian tube after *C. trachomatis* infection and for how they participate in the development of tubal EP.​
3. The data may provide a new diagnostic opportunity for tubal EP in women with *C. trachomatis* infection.

## 1 Data

**Fig. 1:**
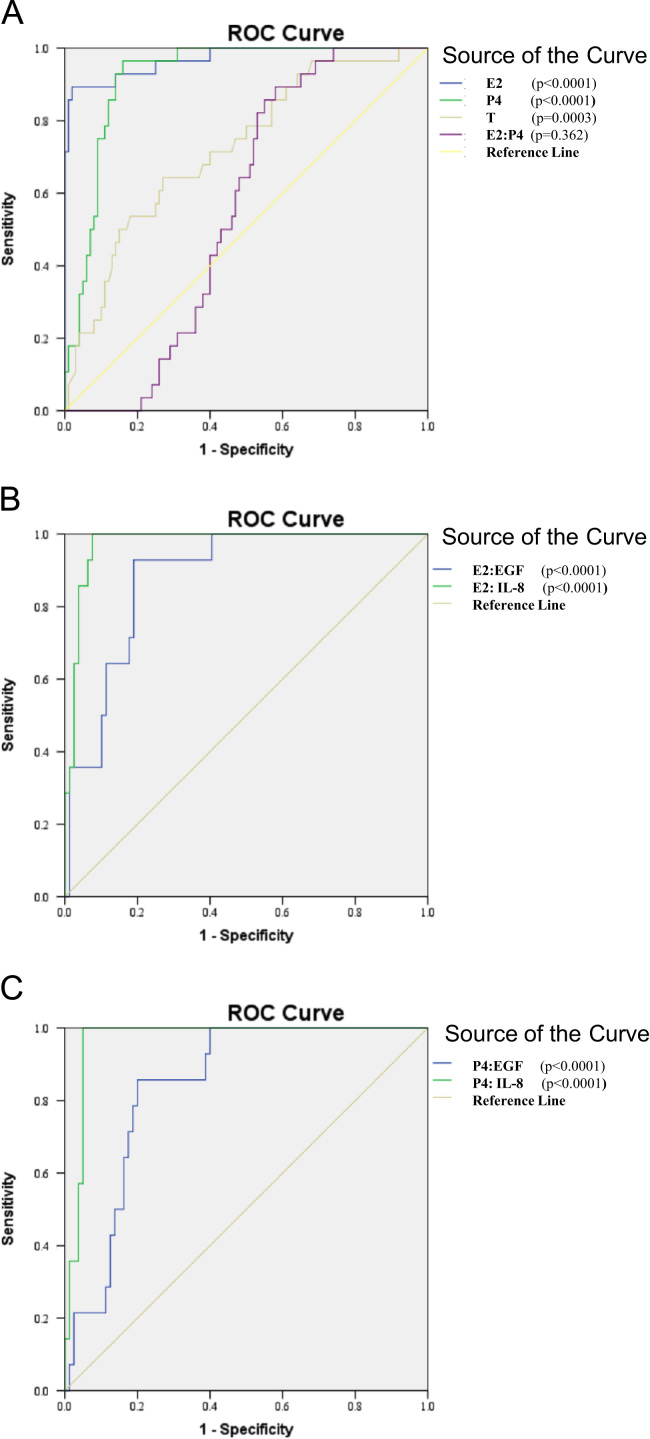
(A) E2, P4, T levels and the E2:P4 ratio as diagnostic tests for women with tubal EP were assessed by the receiver operating curve (ROC) test. ROC analysis was performed with SPSS version 16.0 for Windows, and statistical significance (*P*-value) is indicated. The area under the curve was 0.97 (95% CI: 0.94–1.00) for E2, 0.92 (95% CI: 0.88–0.97) for P4, 0.72 (95% CI: 0.62–0.83) for T, and 0.56 (95% CI: 0.49–0.65) for the E2:P4 ratio. (B) The E2:EGF and E2:IL-8 ratio as diagnostic tests for women with tubal EP were assessed by the ROC test. The area under the curve was 0.88 (95% CI: 0.81–0.96) for the E2:EGF ratio, and 0.97 (95% CI: 0.95–1.00) for the E2:IL-8. (C) The P4:EGF and P4:IL-8 ratio as diagnostic tests for women with tubal EP were assessed by the ROC test. The area under the curve was 0.84 (95% CI: 0.76–0.93) for the P4:EGF ratio, and 0.97 (95% CI: 0.93–1.00) for the P4:IL-8 ratio.

1. The demographics and laboratory characteristics of the normal menstrual cycle women and those with IUP and EP are shown in Table 1. The rates of *C. trachomatis* infection in the women with tubal EP, IUP, and no pregnancy were 86.11%, 60.00%, and 55.40%, respectively (Table 2).
2. Although there was no significant difference in IL-8 levels between IUP and non-pregnancy, IL-8 levels were significantly higher in *C. trachomatis*-positive women with tubal EP than in women with IUP and in non-pregnant women regardless of *C. trachomatis* infection status (Table 2). The diagnostic accuracy of the various parameters (E2, P4, T levels and the E2:P4, E2:EGF, E2:IL-8, P4:EGF, and P4:IL-8 ratio) in the tubal EP and early IUP groups was evaluated by ROC analysis (Fig. 1). The ROC analysis showed that the IL-8 level had excellent discriminative validity in positively identifying tubal EP (concomitant with *C. trachomatis* infection) from IUP and non-pregnant conditions regardless of *C. trachomatis* infection (Fig. 1).
3. An increase in IL-1β levels and a decrease in IL-10 levels were observed in *C. trachomatis*-positive women with tubal EP compared to *C. trachomatis*-positive women with IUP and *C. trachomatis*-positive non-pregnant women (Table 2).
4. There were no significant differences in IL-4, IL-6, IL-7, IL-8, TNFα, or IFN-γ levels among *C. trachomatis*-positive or negative women under pregnant and non-pregnant conditions (Table 2).
5. We also found that individual measurements of serum EGF levels were strongly related to early pregnancy outcomes for women with tubal EP and IUP (Table 2).

## 2 Experimental design, materials and methods

### 2.1 Ethics statement

This study was approved by the Ethics Committees of the Obstetrics and Gynecology Hospital and Shanghai Medical College, Fudan University, China. All participants provided informed consent.

### 2.2 Experimental design and sample collection

All participants underwent clinical examination at the Obstetrics and Gynecology Hospital of Fudan University, Shanghai, China. Clinical work-up included menstrual history as well as current cycle length and menstrual regularity. Exclusion criteria included use of estrogen- or progestin-containing medication within three months of the study, past EP, any gynecological pathology (e.g., endometriosis, fibroids, or any operation to the gynecological organs), infection, and smoking. All the patients whose pregnancies resulted from assisted reproductive technologies were also excluded from the study. Blood samples were collected into Sarstedt evacuated tubes without anticoagulant. All blood samples were centrifuged at 1000×*g* for 15 min, and the serum was stored at –80° C until batch analyses. The present study included non-pregnant and pregnant women (total *n*=225) who were subdivided into the following groups:

1. Group 1. The different stages of the menstrual cycle in non-pregnant women (*n*=139) were studied. Blood samples were collected at the scheduled visits during their menstrual cycle. Menstrual cycle day was established using the criteria reported by Noyes et al. [1]. Sample dating characterized the samples as coming from the proliferative (days 1–14 of the cycle), early secretive (days 15–18), mid-secretive (days 19–23), and late secretive (days 24–28) phases of the menstrual cycle. Regular menstrual cycles were defined as an average cycle length of 26–30 days, with no more variation than±3 days from the average. Transabdominal ultrasonography was also performed to assess ovarian volume and uterine thickness.
2. Group 2. This group comprised healthy IUP women (*n*=50). Blood samples were collected at the scheduled visits during their IUP, and on days 1–3 after spontaneous labor and vaginal delivery. The diagnosis of a normal IUP was made upon the observation of an intrauterine gestational sac or a live embryo on the transvaginal or transabdominal ultrasound scan. Only women with normally progressing pregnancies were studied during their visits to the prenatal clinic during the early stage of their IUP.
3. Group 3. In this group, women with tubal EP (*n*=36) were studied before and after tubal surgery and matched to a subgroup of women with early IUP. A full medical history was documented, and clinical examination was carried out by the attending physician. Transvaginal ultrasonography was performed and the serum β-hCG levels were analyzed in patients at the time of their first clinical presentation. Blood samples were collected from patients at the time of surgery or 2–3 days after surgery. None of the women undergoing surgical management of EP presented with acute hemodynamic shock. Women with EP were diagnosed during laparoscopy and on histological examination of the surgical specimens.

### 2.3 Main outcome measures

All sera were stored at –80 °C before performing the assays, and aliquots that had not been previously thawed were used in the present study. Samples were tested in duplicate and analyzed individually. Radioimmunoassays (RIA) were performed at Beijing Free Co. (China), and enzyme-linked immunoassays (ELISA) were performed at the Department of Integrative Medicine and Neurobiology, Shanghai Medical College, Fudan University (China). The averages of the duplicate readings for each standard, control, and individual samples were used for the analyses.

1. 17β-estradiol (E2), progesterone (P4), testosterone (T), and β-hCG assays: Serum E2 (with an assay sensitivity less than 5.0 pg/mL, an intra-assay coefficient of variation (CV) of 10.0%, and an interassay CV of 15.2%), P4 (with an assay sensitivity less than 5.0 ng/mL, an intra-assay CV of 5.0%, and an interassay CV of 10.0%), T (with an assay sensitivity less than 0.1 ng/mL, an intra-assay CV of 8.0%, and an interassay CV of 15.0%), and β-hCG (with an assay sensitivity less than 10.0 mIU/mL, an intra-assay CV of 5.0%, and an interassay CV of 10.0%) levels were measured by competitive RIA (^125^I-Kit, Beijing Free Co.) using direct-coated tube technology. E2, P4, T, and β-hCG were labeled with ^125^I as the tracer, and known quantities of unlabeled E2, P4, T, and β-hCG were used to construct standard curves. The concentrations used for the standard curves were 0–4000 pg/mL for E2, 0–100 ng/mL for P4, 0–10 ng/mL for T, and 0–1600 mIU/mL for β-hCG.
2. Human cytokine magnetic bead array: The levels of cytokines and growth factors were detected by BioPlex instrument (Bio-rad Hercules, CA) using the high sensitivity human cytokine magnetic bead kit (HSCYTMAG-60SK) and angiogenesis/growth factor magnetic bead panel 1 kit (HAGP1MAG-12K) kits (Merck Millipore Corporation, Billerica, MA) according to the manufacture’s instruction. The intra-assay and inter-assay % CV were indicated in Table 3.

### 2.4 Statistical analysis

Numerical, grouped results are expressed as the mean±SEM. In all analyses, a P value less than 0.05 was considered statistically significant. Statistical analysis was performed using SPSS version 16.0 for Windows (SPSS Inc., Chicago, IL). Comparison among the various groups was performed using nonparametric tests (Kruskal–Wallis followed by multiple comparison procedures according to Dunn’s method) because our variables did not have a normal distribution. Correlation between variables was performed using Spearman’s analysis. The specificity and sensitivity of the various assays as diagnostic tests were assessed using receiver operating characteristic (ROC) curve analysis [2]. As opposed to accuracy, sensitivity and specificity are not dependent on the prevalence of the disease in the sample. Thus, ROC curve analysis provides a description of disease detectability that is independent of both disease prevalence and decision threshold effects. For ROC analysis, women with EP were considered affected, and IUP as unaffected. ROC curves were constructed by plotting the sensitivity (true-positive) on the ordinate as a function of the complement of specificity (false-positive) for all possible cut-off values of the diagnostic test [3]. Greater deviation toward the left upper corner of the curve indicates better detection of tubal EP.

## Competing interests

The authors indicate no potential conflicts of interest.

## Figures and Tables

**Table 1:** Patient characteristics and hormonal profiles.

	n	**Age**	**GA**	**E2**	**P4**	**E2 / P4**	**T**	**β-hCG**	**Chlamydia IgG** positive (%)
(years)	(days)	(pg/mL)	(ng/mL)		(ng/mL)	(mIU/mL)
**Menstrual cycle phase**									55.40%
Proliferative	44	25.98±0.58	–	37.16±4.22	0.94±0.12⁎⁎⁎	0.0610± 0.0096	0.24±0.03	–
Early secretive	25	24.48±0.48	–	41.17±7.49	2.96±0.65	0.0416±0.0152	0.32±0.06	–
Mid secretive	37	24.70±0.34	–	43.59±7.16	5.19±0.72	0.0513±0.0298	0.26±0.05	–
Late secretive	33	24.09±0.36	–	34.01±8.37	2.83±0.66	0.0479±0.0160	0.39±0.07	–
**Early IUP**	50	27.02±0.67	51.30±0.81	227.20±13.73⁎⁎⁎	10.47±0.50⁎⁎⁎	0.0234±0.0015	0.51±0.05⁎⁎	16765.21±582.99	60.00%
**EP**	36	27.72±0.67	47.94±2.26	61.03± 13.13^a^	6.00±0.80^a^	0.0150±0.0035	0.44±0.05	3098.19±1002.64^a^	86.11%

GA, gestational age; IUP, intrauterine pregnancy; EP, ectopic pregnancy.

Data are presented as mean ±SEM. Statistical analysis was performed using SPSS version 19.0 for Windows (SPSS Inc., Chicago, IL).

A nonparametric, unpaired test (Kruskal–Wallis test) followed by Dunnett׳s *post hoc* test was used for comparison of continuous variables.

⁎⁎ *p*<0.01.

⁎⁎⁎ *p*<0.001 *vs.* the mid secretive phase of the endometrial cycle.

a *p*<0.001 *vs.* the early IUP.

**Table 2:** Patient cytokine, IFNγ, TNFα and EGF levels.

	**Menstrual cycle phase**								**Early IUP**		**EP**	
	Proliferative		Early secretive		Mid secretive		Late secretive	
	C ( +)	C (−)	C (+)	C (−)	C (+)	C (−)	C (+)	C (−)	C (+)	C (−)	C (+)	C (−)
	(*n*=32)	(*n*=12)	(*n*=19)	(*n*=6)	(*n*=16)	(*n*=21)	(*n*=10)	(*n*=22)	(*n*=30)	(*n*=20)	(*n*=31)	(*n*=5)
IL-1β	12.12±7.88	0.62±0.25	3.99±2.21	1.05±1.05	6.64±2.59	1.05±0.60	1.44±0.57	0.70±0.34	0.91±0.44	1.73±0.87	5.29±2.08	6.25±2.79
IL-4	29.98±17.89	4.39±1.76	21.71±8.26	U.D.	9.82±6.07	18.06±9.26	1.38±1.38	19.50±10.66	8.41±5.37	2.13±1.05	8.41±3.01	38.98±19.48
IL-6	4.64±1.64	1.97±1.01	5.58±3.36	1.73±0.70	6.66±3.95	4.13±1.19	1.59±0.61	3.64±1.46	9.72±5.86	2.85±1.20	4.39±1.20	8.66±0.91
IL-7	7.88±1.19	10.72±3.55	4.96±0.77	3.98±1.62	14.30±9.43	8.31±3.74	5.07±1.96	3.97±0.72	15.32±12.00	5.43±1.48	5.06±0.88	11.02±4.52
IL-8	18.14±3.93	15.13±4.54	21.02±4.11	30.05±13.57	19.37±3.41	37.64±13.96	15.49±3.77	32.69±10.43	6.03±1.12	7.06±1.57	309.05±163.91⁎⁎, a	53.76±30.73
IL-10	40.00±30.35	80.72±68.14	10.90±3.42	11.71±7.31	49.42±40.10	9.66±2.14	6.70±2.80	14.71±3.82	40.25±30.91	9.36±3.89	15.92±6.95	25.79±13.21
IFNγ	13.76±3.01	37.29±32.71	43.88±33.25	7.36±2.88	12.61±3.36	23.89±11.28	5.75±1.69	18.41±9.40	6.64±4.06	4.77±2.32	3.94±0.86	22.83±13.65
TNFα	13.80±4.28	9.79±4.26	8.89±3.04	4.33±0.72	8.42±2.02	5.86±0.79	7.15±1.31	4.90±0.81	3.82±0.96	3.19±0.72	6.89±1.82	10.77±2.65
EGF	288.29±23.59	280.47±33.24	343.31±37.18	367.91±64.03	305.10±45.50	299.69±38.64	393.10±48.15	329.70±28.29	557.49±48.35⁎⁎⁎	558.87±53.45	553.60±71.60⁎⁎⁎	396.22±60.06

IUP, intrauterine pregnancy; EP, ectopic pregnancy; C (+), *Chlamydia trachomatis* IgG positive; C (−), *Chlamydia trachomatis* IgG negative. U.D., under the detection.

The units of all cytokines and factors are pg/mL.

Data are presented as mean±SEM. Statistical analysis was performed using SPSS version 20.0 for Windows (SPSS Inc., Chicago, IL).

A nonparametric, unpaired test (Kruskal–Wallis test) followed by Dunnett׳s *post hoc* test was used for comparison of continuous variables.

⁎⁎ *p*<0.01.

⁎⁎⁎ *p*<0.001 *vs.* mid secretive phase of menstrual cycle C (+).

a *p*<0.001 *vs.* the early IUP C(+).

**Table 3:** The intra-assay and inter-assay % CV for the human cytokine magnetic bead kit and angiogenesis/growth factor magnetic bead panel 1 kit.

**Analyze**	**Intra-assay % CV**	**Inter-assay % CV**
IL-1β	9.3	9.6
IL-4	10.8	15.6
IL-6	11.9	10.3
IL-7	10.9	15.6
IL-8	3.0	7.1
IL-10	10.0	15.2
IFNγ	10.6	9.5
TNFα	10.6	9.8
EGF	3.2	6.8
*Chlamydia* IgG	3.7	2.8

*Chlamydia* IgG was detected by *Chlamydia trachomatis* IgG human ELISA kit.

Positive: >11 NTU; Negative: <11 NTU.

NTU: Abcam units.
